# Effectiveness of* Coelatura aegyptiaca* Extract Combination with Atorvastatin on Experimentally Induced Hyperlipidemia in Rats

**DOI:** 10.1155/2019/9726137

**Published:** 2019-01-01

**Authors:** Ayman Saber Mohamed, Walaa Mohammed Ibrahim, Nashwah Ismail Zaki, Sara Bayoumi Ali, Amel M. Soliman

**Affiliations:** ^1^Lecturer of Physiology, Zoology Department, Faculty of Science, Cairo University, 12613 Giza, Egypt; ^2^M.Sc. Student, Zoology Department, Faculty of Science, Cairo University, Egypt; ^3^Professor of Physiology, Physiology Department, National Organization for Drug Control and Research, Egypt; ^4^Professor of Physiology, Zoology Department, Faculty of Science, Cairo University, Egypt

## Abstract

**Background:**

The present study aimed to assess the effectiveness of clam extract in combination with atorvastatin against experimentally hyperlipidemia in rats.

**Method:**

Forty male rats were divided into 5 groups (8 rats /group): control, high fat diet (HFD), atorvastatin (AROR), clam extract (CE), and ATOR + CE.

**Results:**

The treatments with ATOR and /or CE significantly reduced the body weight gain, AST, ALT, ALP, TL, TC, TG, LDL-C, urea, creatinine, and uric acid levels while they increased total proteins, albumin, and HDL-C. The treatment with ATOR only did not cause any significant change in CK and MDA along with antioxidant system, while the treatment with CE alone or with ATOR significantly decreased CK and MDA accompanied by improving the antioxidant system.

**Conclusion:**

Combination of CE extract with atorvastatin improved the hyperlipidemic efficacy and reduced undesirable side effects especially on muscle.

## 1. Introduction

Obesity is one of the highest public health epidemics of the 21st century with about 2 billion adults worldwide currently classified as being overweight or obese [1]. It is considered a serious chronic disease that increases the risk of numerous comorbidities including metabolic syndrome, cardiovascular disease, and cancer along with increases risk of mortality leading some to suggest this represents accelerated aging [2].

There is significant evidence that elevated oxidative stress mediates the progression of many of the comorbidities associated with obesity [2]. Epidemiological, clinical, and animal studies have reported the role of oxidative stress in the pathogenesis of obesity and its related risk factors [3]. The increase of adipose tissue is linked with an increased production of reactive oxygen species (ROS) in both human and rodent models through various mechanisms [4]. In obese and type II diabetics, insulin resistance causes impairment of the endocrine function of perivascular adipose tissue, an imbalance in the secretion of vasoconstrictor and vasodilator molecules, and increasing of the production of ROS [5]. In addition, the hypertriglyceridemia may contribute to the alteration in the oxidant-antioxidant balance, suggesting that an increase in the bioavailability of free fatty acids can increase lipid peroxidation [6]. Feeding of high fat diet (HFD) to rats was proved to be a useful model of putative effects of dietary fat in humans [7]. Rat models are therefore useful tools for inducing obesity as they will readily gain weight when HFD [8].

Statins consider the standard drug in the treatment of hypercholesterolemia and the management of patients with increased cardiovascular disease risk [9]. Statins are well tolerated, but are associated with many side effects [9]. It was observed that statin therapy decreases antioxidant status by inhibition of Coenzyme Q10 and the subsequent mitochondrial energetic metabolism disorder may lead to myopathy [10, 11]. Nearly all of the statin drugs are associated with musculoskeletal side effects [12].

Many natural products have hypolipidemic effects and can be used in treatment of obesity as alkaloid extract of the cyanobacterium Spirulina platensis [13], echinochrome extracted from sea urchin [14], and freshwater clams extract [15]. Egyptian freshwater clam (*Coelatura aegyptiaca*) is a Molluscan Bivalve belonging to Unionidae common in the Egyptian Nile River [16]. Many in vitro and in vivo studies have found that freshwater clams possess many medical and biological effects, including antitumorigenic properties [17], hepatoprotective [18], and cholesterol-lowering [15, 19]. Therefore, the present study aimed to assess the effectiveness of* Coelatura aegyptiaca* extract in combination with atorvastatin against experimentally hyperlipidemia in rats.

## 2. Materials and Methods

### 2.1. Chemicals and Reagents

Atorvastatin powder and Carboxymethyl cellulose (CMC) were purchased from Sigma-Aldrich (St. Louis, MO, USA). Kits of biochemical parameter were purchased from Biodiagnostic Company (Dokki, Giza, Egypt).

### 2.2. Collection and Preparation of Clam Extract (CE)

Freshwater clams (*Coelatura aegyptiaca*) were collected from the River Nile at Giza Governorate, Egypt. Whole bodies away from the shells were stored at -20°C until needed. After thawing, CE was prepared as follows: whole bodies (1 kg) were coarsely chopped and homogenized with a mixer. The homogenate was extracted in a boiler with 1 liter of distilled water for 3 hrs and allowed to cool to room temperature. After filtration, the filtrate obtained was then concentrated and dried using a lyophilizer (LABCONCO lyophilizer, shell freeze system, USA), the dose chosen according to Soliman [15].

### 2.3. Experimental Animals

Male albino Wistar rats (*Rattus norvegicus*) weighing 140±10 g were used in this study. The rats were obtained from the National Research Center (NRC, Dokki, Giza). Rats were housed in a temperature and humidity controlled environment and given food and water ad libitum.

### 2.4. Ethical Consideration and Field Study

Experimental protocols and procedures used in this study were approved by the Cairo University, Faculty of Science, Institutional Animal Care, and Use Committee (IACUC) (Egypt) (CUFS/F/33/15). All the experimental procedures were carried out in accordance with international guidelines for the care and use of laboratory animals.

### 2.5. Induction of Hyperlipidemia

The rats were fed a high fat diet (HFD) with energy of 5.3 kcal/g, comprising 40% calories from fat, 17% from protein, and 43% from carbohydrate for 4 weeks according to a modification of the protocols of Reed et al. [20]. The diet consists of a mixture of 68% normal rat chow pellet (purchased from El Gomhorya Company, Ismailia, Egypt), 6% corn oil, 20% milk powder, and 6% ghee, while the normal rat chow diet contains 3.06 kcal/g with 21% from protein, 48.8% from carbohydrate and 3% from fat.

### 2.6. Experimental Design

After one week of acclimatization, forty rats were divided into five groups (8 rats /group).

*Control Group*. After 4 weeks of normal diets feeding, the rats were given orally 1 ml of 0.5% CMC, daily for 16 days.

*HFD Group*. The rats were fed HFD for 4 weeks and then were given orally 1 ml of 0.5% CMC, daily for 16 days.

*Atorvastatin Group (ATOR)*. After 4 weeks of HFD feeding, the rats were given 1 ml of ATOR (80 mg/kg in 0.5% CMC, orally) daily for 16 days [21].

*Clam Extract (CE) Group*. After 4 weeks of HFD feeding, the rats were given 1 ml of CE (100 mg/kg in 0.5% CMC, orally) daily for 16 days [15].

*ATOR + CE Group*. After 4 weeks of HFD feeding, the rats were given 1 ml of ATOR (80 mg/kg in 5% CMC, orally) and after 1 hour were given 1 ml CE (100 mg/kg in 5% CMC, orally) daily for 16 days.

### 2.7. Body Weight Gain, Tissue Weight and Indices

Body weight was measured weekly and body weight change was measured from difference between final body weight and initial body weight. Liver and kidney weighed and their indices were calculated (Tissue  index = (tissue  weight/body  weight) × 100 ).

### 2.8. Animal Handling and Specimen Collection

After the end of all experiments, the rats were fully anesthetized with 3% sodium pentobarbital, and the chest was opened. A needle was inserted through the diaphragm and into the heart. Negative pressure was gently applied once the heart had been punctured, and the needle was repositioned as required until blood flowed into the syringe. The blood collected from the rats was separated by centrifugation (3000 rpm, 15 min) to obtain sera which were stored at −80°C for the biochemical measurements.

Liver, kidney, and skeletal muscles were removed and were immediately blotted using a filter paper to remove traces of blood. Parts of the tissues were stored at -80°C for biochemical analyses and other parts were suspended in 10% formal saline for fixation preparatory to histopathological examination.

### 2.9. Tissues Homogenate Preparation

Liver, kidney, and skeletal muscles tissues were homogenized (10% w/v) in ice-cold 0.1 M Tris–HCl buffers (pH 7.4). The homogenates were centrifuged at 3000 rpm for 15 min at 4°C and the resultant supernatant was used for the oxidative stress analyses.

### 2.10. Histopathological Examination

The liver, kidney, and skeletal muscles tissues were sectioned at a thickness of 4-5 *μ*m and stained with hematoxylin and eosin (H&E) according to Bancrof and Stevens [22], as routine procedures for histopathological examination. All tissue sections were assessed under light microscopy independently by two investigators in a blinded manner. Severity of tissue injury was scored 0, absent; 1, few; 2, mild; 3, moderate; and 4, severe for each of the parameters. The parameters are cells vacuolization, nuclear fragmentation, and cell necrosis [23]. The histology injury scores were expressed as the sum of the individual scores. Ten high-power fields per sample were scored and averaged to represent each animal.

### 2.11. Biochemical Analyses

The serum aspartate aminotransferase (AST) and alanine aminotransferase (ALT) were estimated by the method of Reitman and Frankel [24]; serum alkaline phosphatase (ALP) [25], serum total protein [26], serum albumin [27], serum total lipids (TL) [28], serum triglycerides (TG) [29], serum total cholesterol (TC) [30], serum low density lipoprotein (LDL-C) [31] and serum high density lipoprotein (HDL-C) [32], serum creatinine [33], serum urea [34], serum uric acid [35], and creatine kinase (CK) [36] were determined according to the manufacturer's instructions using Spectrum Diagnostics and Biodiagnostic kits (Giza, Egypt).

MDA level is an index of lipid peroxidation and it was estimated by Ohkawa et al. [37], glutathione reduced (GSH) [38], glutathione-S-transferase (GST) [39], and catalase [40] were determined in the liver, kidney, and muscle homogenates supernatant according to the manufactures instructions using Biodiagnostic kits (Giza, Egypt).

### 2.12. Statistical Analysis

Values were expressed as means ± SE. The comparisons within groups were evaluated utilizing one-way analysis of variance (ANOVA) with Duncan post hoc test used to compare the group means and p < 0.05 considered statistically significant. SPSS, for Windows (version 15.0), was used for the statistical analysis.

## 3. Results

The body weight gain, liver weight, kidney weight, and their indices increased significantly (p< 0.05) in HFD group as compared with control group, while the treatments with ATOR and / or CE significantly (p< 0.05) reduced theses parameters as compared to HFD group (Table 1).

Our data shown in Table 2 represented that the HFD caused significant increases (p< 0.05) in AST, ALT, ALP, TL, TC, TG, LDL-C, urea, creatinine, uric acid, and CK levels while total proteins, albumin, and HDL-C concentrations decreased significantly (p< 0.05) as compared to control group. On the other hand, the treatment with ATOR and/or CE showed significant decreases (p< 0.05) in AST, ALT, ALP, TL, TC, TG, LDL-C, urea, creatinine, and uric acid levels whereas total proteins, albumin, and HDL-C concentrations increased significantly (p< 0.05) as compared to HFD group. In addition, all treatment groups except ATOR showed significant decrease (p< 0.05) in CK activity as compared to HFD group (Table 2).

Data recorded in Table 3 demonstrated that HFD feeding caused significant increase (p< 0.05) in liver, kidney, and muscle MDA concentration while GSH, GST, and CAT levels decreased significantly (p< 0.05) as compared to control groups. Also the treatment with ATOR showed nonsignificant changes (p > 0.05) in these parameters as compared to HFD group. On the other hand, administration of CE alone or combined with ATOR causes significant decrease (p< 0.05) in MDA and significant increases (p< 0.05) in GSH, GST, and CAT levels as compared to HFD group (Table 3).

### 3.1. Histopathological Examination of Liver

**Figure 1:**
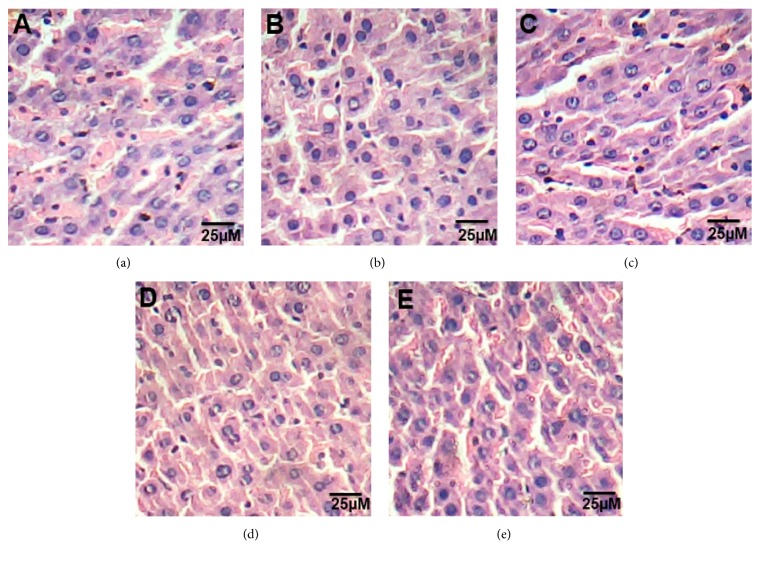
Effect of CE and ATOR on liver of hyperlipidemic rats. Photomicrograph of hematoxylin and eosin stained liver sections from (a) Control rats group (Score 0), (b) HFD group (Score 3), (c) ATOR group (Score 1), (d) CE group (Score 1), and (e) ATOR + CE group (Score 1) (H&E × 400).

The liver of control rats is formed of the classic hepatic lobules. Blood sinusoids were seen separating the cords of the liver cells and lined by flattened endothelial cells and von Kupffer cells (Figure 1(a)). The histology of liver sections obtained from HFD rats showed clear diffused hepatic steatosis with the loss of usual concentric arrangements of hepatocytes, fatty changes with necrosis, and congested sinusoids (Figure 1(b)). Liver sections of ATOR, CE, and ATOR + CE rats showed moderate to mild degenerated changes in hepatocytes and clear improvement in the hepatic architecture compared to HFD group (Figures 1(c), 1(d), and 1(e)).

### 3.2. Histopathological Examination of Kidney

**Figure 2:**
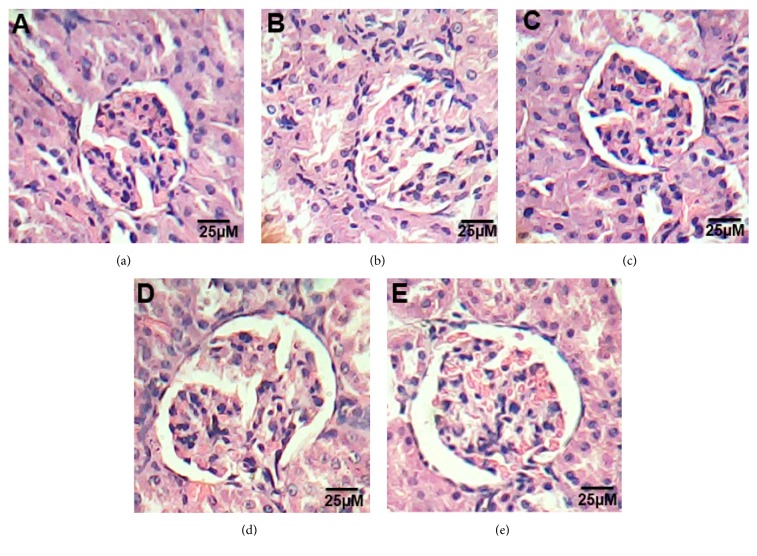
Effect of CE and ATOR on kidney of hyperlipidemic rats. Photomicrograph of hematoxylin and eosin stained kidney sections from (a) Control rats group (Score 0), (b) HFD group (Score 4), (c) ATOR group (Score 2), (d) CE group (Score 1), and (e) ATOR + CE group (Score 1) (H&E × 400).

Microscopic examination of kidney sections of control groups showed normal appearance of the tissue where glomeruli appear as dense tufts of capillaries enclosed in the outer layer of Bowman capsules. Numerous renal tubules were observed (Figure 2(a)). Kidney section of HFD rats showed swelling and proliferation in the endothelial cells lining the glomerular tuft of the glomeruli as well as degeneration in the epithelial cells lining the tubules, brush border loss necrosis, and deformed renal tissue architecture (Figure 2(a)). On the other hand, treated groups with ATOR, CE, and ATOR + CE showed mild degeneration in renal tissue architecture, as compared to HFD groups (Figures 2(c), 2(d), and 2(e)).

### 3.3. Histopathological Examination of Muscle

**Figure 3:**
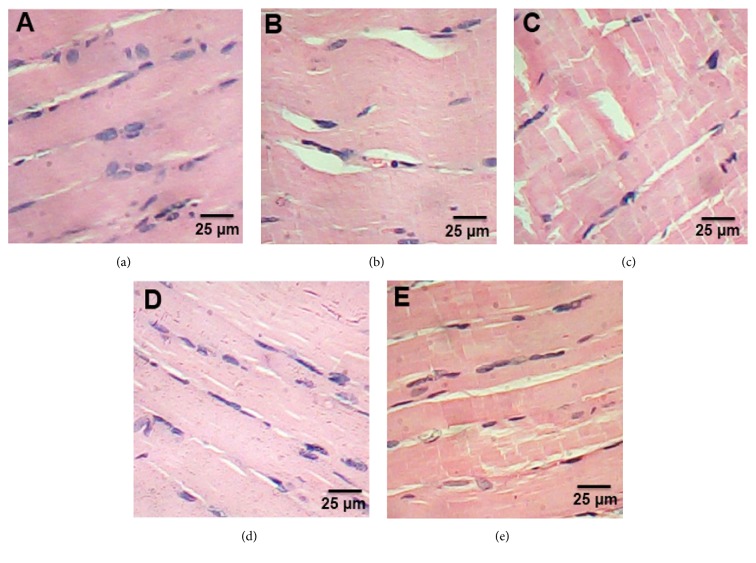
Effect of CE and ATOR on muscle of hyperlipidemic rats. Photomicrograph of hematoxylin and eosin stained muscle sections from (a) Control rats group (Score 0), (b) HFD group (Score 4), (c) ATOR group (Score 3), (d) CE group (Score 1), and (e) ATOR + CE group (Score 1) (H&E × 400).

Microscopic examination of extensor digitorum longus muscle of control group revealed the normal structure of skeletal muscle (Figure 3(a)). Muscle section in HFD group showed splitting of the myofibers, peripheral elongated nuclei disappeared, and striation is lost in some muscle fibers (Figure 3(b)). Muscle section of ATOR group showed mild focal changes and irregular variation in muscle fibers size. Nuclei were internal in position instead of peripheral and some appeared rounded in shape instead of oval. Splitting of some fibers was also observed which appeared as a transverse invagination or complete separation (Figure 3(c)). CE and CE + ATOR treatment of rats was associated with normalization of skeletal muscle features which were similar to that of untreated controls (Figures 3(d) and 3(e)) (H & E x 400).

## 4. Discussion

Obesity is the current medical as well social problem, leading to increasing morbidity and mortality. The main risk factors of hyperlipidemia are associated with atherosclerosis, which predisposes ischemic heart disease and cerebrovascular disease [41]. The present study amid to study the hypolipidemic activity of clam extract (CE) alone or with statin drug against HFD-induced obesity in rats.

Our results revealed that the body weight gains of the rats in the HFD group were significantly higher than that in the control group, and the weight gains in the ATOR, CE, and ATOR+ CE groups were normalized to or lower than that in the HFD group. The consumption of a HFD facilitates the development of a positive energy balance and leads to an increase in visceral fat deposition [42]. However, our results confirm efficacy of ATOR and/or CE in reducing the body weight gains of obese rats.

Liver biomarkers enzymes “AST, ALT, ALP” are monitoring the liver structural integrity and damage [43]. In the current study, administration of HFD caused increases in concentrations of ALT, AST, and ALP enzymes and decreased total proteins and albumin concentrations. The increase of these liver enzymes values may be indicative of some liver impairment or possibly damage [44], which confirmed by the histopathological investigation of liver sections. Liver damage resulting from underlying cellular death is often associated with obesity [45]. Instead, the treatment with ATOR and/or CE decreased the concentrations of these enzymes and increased total proteins and albumin concentrations which may be related to the hypolipidemic activity of them [9, 19, 46]. The histopathological examination of liver confirms the ameliorative effect of the treatments.

In the current study the administration of HDF resulted in dyslipidemic changes as illustrated by increasing TL, TG, TC, LDL-C, and low level of HDL-C. Dyslipidemic changes occurring in obesity may be due increased influx of excess nonesterified fatty acids into the liver [47]. While the hypolipidemic activity of ATOR and/or CE resulting in the decreasing in TL, TG, TC, LDL-C, and high level of HDL-C. The hypolipidemic mechanism of statin is through the competitive, reversible inhibition of HMG-CoA reductase, the rate-limiting step in cholesterol biosynthesis [48], while CE improved cholesterol level through the gene expression of CYP7A1, involved in the stimulation of bile acid synthesis and fecal sterol excretion [19]. Additionally CE reduces triglycerides through the suppression of gene expression related to lipogenesis [46].

Abnormalities in lipid metabolism appear to play a pathogenic role in development of renal diseases [49, 50]. In the present study, HFD-induced renal injury indicated by elevation of urea, creatinine, and uric acid concentration additional to the histopathological investigation. HFD leads to an altered balance between renal lipogenesis and lipolysis, subsequent renal accumulation of lipid, and renal injury [51]. On the other hand the treatment with ATOR and / or CE improved the renal biomarkers values which my be related to the hypolipidemic activity of them [9, 19, 46].

Obesity is related to skeletal muscle loss and dysfunction and the development of muscle atrophy [52]. Also, feeding with a HFD develops the typical features of muscle wasting, such as the loss of muscle mass, weakness, and decreased fiber diameter [53]. Muscle injury in the present study was pronounced in HFD group by significant increase in CK and histopathological investigation. HFD induces skeletal muscle injury through the stimulation of several mechanisms, such as increased oxidative stress, ubiquitin proteasome pathway overactivation, and the generation of myonuclear apoptosis [54]. Unfortunately, the treatment with ATOR alone induced muscle injury in the current study. These facts have supported the idea that statin induced myopathy which could be attributed to decreased Coenzyme Q10 in muscular tissue [55]. However, the treatment with CE alone or with ATOR leads to significant decrease in CK and improvement in the histological sections of rat muscles. The results indicated the ability of CE to reduce the side effects of statin on muscle.

Oxidative stress is produced through increased reactive oxygen species (ROS) and/or decreased antioxidant mechanisms [56]. High fat diet-induced obesity is accompanied by increased tissues oxidative stress, which is characterized by reduction in the antioxidant enzymes activities and glutathione levels that correlate with the increase in MDA levels in most tissues [57]. Our study confirms these results in liver, kidney, and muscle tissues of HFD group. Obesity can cause increased lipid peroxidation via progressive and cumulative cell injury resulting from pressure of the large body weight [57]. Cell injury leads to the release of cytokines “especially tumor necrosis factor alpha (TNF-a)” which produces ROS from the tissues which in turn cause lipid peroxidation [58]. The treatment with ATOR alone also decreases antioxidant status in the liver, kidney, and muscle tissues by inhibition of Coenzyme Q10 [11, 59], since coenzyme Q10 provides energy to the cells and has powerful antioxidant activity [60]. Interestingly the treatment with CE alone or with ATOR improves the antioxidant system and reduced lipid peroxidation which considers the main defect in the treatment with ATOR alone. The antioxidant activity of CE was confirmed by many previous studies [15, 18].

## 5. Conclusion

Combination of CE extract with atorvastatin improved the hyperlipidemic efficacy and reduced undesirable side effects especially on muscle.

## Figures and Tables

**Table 1:** Effect of CE and ATOR on body weight gain, liver weight, kidney weight, liver index, and kidney index of hyperlipidemic rats.

**Parameters**	**Control**	**HFD**	**ATOR**	**CE**	**ATOR + CE**
Wight gain (%)	36.71±4.01^b^	51.10±6.65^c^	25.84±2.65^a^	33.72 ±4.32^b^	24.40 ±1.65^a^
Liver weight (g)	4.90 ± 0.28 ^b^	6.83 ± 0.35^d^	4.99 ± 0.38 ^b^	5.19 ± 0.41 ^c^	4.75 ± 0.29 ^a^
kidney weight (g)	1.02 ± 0.09 ^a^	1.42 ± 0.05^d^	1.16 ± 0.06 ^c^	1.22 ± 0.07^c^	1.08 ± 0.08 ^b^
Liver index	2.35± 0.51 ^a^	5.65± 0.58 ^c^	2.22± 0.48 ^a^	2.92± 0.31 ^b^	2.15± 0.21 ^a^
Kidney index	0.31± 0.02 ^a^	0.51± 0.03 ^c^	0.29± 0.02 ^a^	0.34± 0.02 ^b^	0.27± 0.04 ^a^

Values are means ± SE (n = 6 per group). Each value not sharing a common letter superscript is significantly different (P <0.05).

**Table 2:** Effect of CE and ATOR on serum biomarkers analysis of hyperlipidemic rats.

**Parameters**	**Control**	**HFD**	**ATOR**	**CE**	**ATOR + CE**
AST (IU/ml)	118.50±2.77^a^	220.00±3.09^d^	139.83±2.04^b^	154.83±2.15^c^	121.3±31.49^a^
ALT (IU/ml)	33.00±1.06^a^	65.33±1.52^d^	41.50±1.48^b^	47.17±1.70^c^	37.67±2.03^a^
ALP (IU/L)	66.83±2.46^a^	142.67±2.70^c^	78.50±1.95^b^	80.17±2.94^b^	70.17±1.66^a^
Albumin (g/dL)	4.02±0.12^d^	3.15±0.16^a^	3.40±0.07^b^	3.32±0.10^b^	3.58±0.09^c^
T. Protein (g/dL)	7.13±0.13^d^	5.15±0.13^a^	5.90±0.04^b^	5.53±0.08^b^	6.23±0.20^c^
TL (mg/dl)	59.67±6.15^a^	293.83±35.88^d^	125.67±9.88^b^	207.17±3.99^c^	120.00±6.26^b^
TC (mg/dl)	27.37±1.17^a^	59.55±2.81^d^	34.07±1.98^b^	44.20±0.54^c^	29.42±1.28^a^
TG (mg/dL)	15.32±1.16^a^	56.47±6.20^c^	23.40±1.44^b^	30.03±2.73^b^	19.82±1.47^a^
HDL-C (mg/dL)	41.17±2.21^b^	25.00±2.45^a^	42.67±0.99^b^	37.67±1.28^c^	40.67±1.80^b^
LDL-C (mg/dL)	14.53±1.85^a^	46.87±1.55^d^	22.62±1.79^b^	32.80±1.21^c^	20.80±0.78^b^
Urea (mg/dl)	17.97±1.00^a^	46.62±1.57^d^	41.62±1.96^c^	40.65±1.34^c^	32.18±0.96^b^
Creatinine (mg/dl)	0.48±0.02^a^	0.68±0.03^c^	0.60±0.02^b^	0.54±0.02^b^	0.49±0.01^a^
Uric acid (mg/dL)	1.17±0.02^a^	2.02±0.07^c^	1.35±0.02^b^	1.33±0.03^b^	1.20±0.04^a^
CK (IU/l)	126.17±16.99^a^	336.83±22.75^c^	294.17±28.87^c^	186.83±14.87^b^	142.33±9.97^a^

Values are means ± SE (n = 6 per group). Each value not sharing a common letter superscript is significantly different (P <0.05).

**Table 3:** Effect of CE and ATOR on oxidative stress biomarkers of hyperlipidemic rats.

**Parameters**		**Control**	**HFD**	**ATOR**	**CE**	**ATOR + CE**
MDA (nmol/g. tissue)	Liver	1.9±0.14^a^	3.58±0.14^b^	3.3±0.06^b^	2.87±0.08^c^	2.1±0.06^a^
	Kidney	6.18±0.26^a^	9.02±0.27^c^	8.87±0.18^c^	7.72±0.51^b^	6.72±0.35^a^
	Muscle	2.25±0.03^a^	3.52±0.19^b^	3.35±0.07^b^	2.15±0.09^a^	2.10±0.06^a^

GSH (mg/g. tissue)	Liver	7.23±0.23^c^	4.65±0.28^a^	5.40±0.31^a^	6.64±0.26^b^	7.10±0.18^c^
	Kidney	15.00±0.58^d^	9.61±0.02^a^	10.23±0.22^a^	11.57±0.46^b^	13.50±0.76^c^
	Muscle	10.85±0.34^b^	8.54±0.44^a^	9.55±0.02^a^	10.02±0.37^b^	10.26±0.10^b^

GST (U/g. tissue)	Liver	0.71±0.02^c^	0.51±0.02^a^	0.55±0.06^a^	0.66±0.03^b^	0.66±0.05^c^
	Kidney	2.36±0.06^c^	1.17±0.07^a^	1.32±0.01^a^	1.93±0.04^b^	2.02±0.05^b^
	Muscle	16.67±1.15^c^	10.50±0.67^a^	12.83±0.75^a^	14.67±0.42b^c^	15.17±0.31^c^

CAT (*μ*mol/g tissue)	Liver	17.8±8.18^b^	10.8±1.66^a^	12.0±2.70^a^	15.6±4.24^b^	16.6±6.73^b^
	Kidney	61.02±2.75^b^	32.93±1.62^a^	36.83±1.21^a^	57.68±1.81^b^	59.52±2.11^b^
	Muscle	25.80±2.35^c^	10.07±1.44^a^	11.55±0.84^a^	16.53±0.86^b^	20.28±1.36^b^

Values are means ± SE (n = 6 per group). Each value not sharing a common letter superscript is significantly different (P <0.05).

## Data Availability

The data used to support the findings of this study are included within the article.
